
JOURNAL INFORMATION
==============================
NLM Title Abbreviation: J Child Orthop
Journal ID: J Child Orthop
NLM Journal ID: 101313582

Journal of Children's Orthopaedics

ISSN: 1863-2521
EISSN: 1863-2548
Publisher: SAGE Publications

ARTICLE INFORMATION
==============================
PMCID: PMC4353814
PMID: 25711754
DOI: 10.1007/s11832-015-0639-y
Article ID: 639
Article version: 1
Subjects: Abstracts

IFPOS 34th Annual Meeting

Electronic publication date: 2015 Feb 25
Print publication date: 2015 Apr
Volume: 9
Issue: Suppl 1
First page: 121
Last page: 132
Copyright: © The Author(s) 2015
License: This article is distributed under the terms of the Creative Commons Attribution License which permits any use, distribution, and reproduction in any medium, provided the original author(s) and the source are credited.
License URL: https://creativecommons.org/licenses/by/4.0/

issue-copyright-statement: © The Author(s) 2015

==============================

**April 16**
IFPOS session
14:20–18:30
VIEUX PORT 300
**IOP1**/14:20–14:25

## Postoperative complications following spinal fusions in children with neuromuscular scoliosis

Tristan Nishnianidze[1], Ilhan A Bayhan[1], Oussama Abousamra[1], Kenneth J Rogers[1], Kirk W Dabney[2], Freeman Miller[2]

[1]NAIDHC, Wilmington DE, United States
[2]NAIDHC, Wilmington, United States

*Purpose:* Cerebral Palsy (CP) patientscommonly have progressive scoliosis that may require posterior spinal fusion (PSF) surgery. The purpose of this study was to define the relationship between preoperative and post-operative clinical factors with postoperative complications and outcomes.

*Methods:* A retrospective review was completed on patients who had PSF for neuromuscular scoliosis between 2004 and 2013 at one institution. Three different instrumentation types were used: unit rod, pedicle screws or hybrid construct implant. Preoperative risk factors included GMFCS, CP diagnosis, nutritional status, mental retardation, severity of spinal deformity, ambulatory status, seizure, physical functional level, speech capacity, feeding ability, cognitive ability and laboratory values. Post-operative clinical course data was collected and included: events in the hospital; complications; and infection types.

*Results*: Three hundred and two children with neuromuscular scoliosis were included with a mean age of 14.6 ± 3.0 years. Mean weight and height were 35.6 kg (+12.7; Z = −2.8 + 4.8) and 138.7 cm (+23.44; Z = −2.8 + (3.3), respectively. GMFCS 5 (205, 67.9 %) was highest in frequency. G-tube and seizure were present in 55 and 58 % of patients. Mean length of days for ICU and total hospitalization were 6 and 16, respectively. Highest frequency instrumentation was Unit rod (205, 66 %). Post-op superficial and deep wound infection rates were 0.027 %, respectively with an average discharge of 28.33 + 21 days. Post-operative complication rates were the following: pneumothorax (16, 5.1 %), post-op reintubation (7, 2.3 %), hyperalimentation (128, 41.2 %), atelectasis (15, 4.8 %), pleural effusion (129, 41.5 %), pneumonia (1, 0.3 %), transfusion reaction (1, 0.3 %), constipation (25, 8.0 %), and pancreatitis (193, 62.1 %). Infection rates post-op were the following: pulmonary (7, 2.3 %), urinary (2, 0.6 %), and septicemia (9, 2.9 %). Average first feed day was 6 + 2. Highest relative risk (rr) factors were calculated for pancreatitis, septicemia, hyperalimentation, deep wound infection, and atelectasis: Pre-op positive weight Z-score > rick constipation (r/r1.559); pre-op seizure > risk infection (rr = 1.224); pre-op seizure > risk pneumothorax (1.417); pre-op seizure > risk atelectasis (rr = 1.39); pre-op seizure > risk hyperalimentation (rr = 2.039); pre-op G-tube > risk pancreatitis (rr = 1.388); pre-op G-tube > risk deep wound infection (rr = 1.715);

*Conclusion:* G-tube and seizure activity (pre-op or post-op) had the highest relative risk value for the primary complications that occurred post-operatively.

*Significance:* Complications after posterior spinal fusion in CP are strongly associated with preoperative positive seizure activity, G-tube feeding and positive weight z-score.

**April 16**
IFPOS session
14:20–18:30
VIEUX PORT 300
**IOP2**/14:25–14:30

## Guided growth of the proximal femur for spastic hip displacement in children with cerebral palsy

Wei Chun Lee[1]

[1]Chang Gung Memorial Hospital, Taoyuan, Taiwan

*Background*: The concept of growth modification by transphyseal screws has been used to correct coxa vara, leg length discrepancy, and ankle valgus deformity. Pilot animal studies showed encouraging results by using one transphyseal screw to produce varus deformity in proximal femur. The purpose of this study is to report the preliminary results of guided growth surgery in treating spastic hip displacement.

*Methods*: This is a case series study including consecutive cases from January 2004 to May 2012 with minimal 2-year follow up. The surgical indications were cerebral palsy children with age from 4 to

10 years, gross motor function classification system level IV or V, and hip displacement in one or both sides. Study outcome was measured by Reimer's migration percentage (MP) and head-shaft angle (HSA).

*Results:* Nine children with 13 spastic displaced hips received guided growth surgery and soft tissue release in the study period. The mean operation age was 6.2 year, and the mean follow up was 45.6 months. Mean MP was improved from pre-operative 52.2 to 45.8 % at post-operative 3 months, 40.3 % at post-operative 1 year and to 37.1 % at post-operative 2 years, and 27.3 % at the latest follow-up. HSA was not changed in the first 3 months, and deceased from 173.3° to 166.4° at 1 year and 162.7° at 2 years. The differences between 3 months and 1 or 2 years were significant in MP and HSA.

*Conclusion*: The immediate change in MP was result of soft tissue release. From post-operative 3 months to 2 years, a reduction of HSA by 10.6° and improvement of MP by 8.7 % was observed. Less surgical dissection, faster recovery of motion, and free from comorbidities in osteotomy make the guided growth surgery a treatment option for coxa valga in spastic hip displacement.

**April 16**
IFPOS session
14:20–18:30
VIEUX PORT 300
**IOP3**/14:35–14:40

## Infection as a complication of intrathecal baclofen treatment in children with cerebral palsy

Ilhan A Bayhan[1], Julie Sees[1], Tristan Nishnianidze[1], Kenneth J Rogers[1], Freeman Miller[1]

[1]NAIDHC, Wilmington DE, United States

*Purpose:* Children with cerebral palsy and spasticity are often managed with intrathecal baclofen treatment (ITB). Complications of ITB include infection at the pump or catheter site and late complications as well as revisions of the pump and catheter because of events such as battery expiration or implant malfunction. The goal of this study is to report the short and longterm incidence, risk factors and treatment outcomes of ITB infections in children.

*Methods*: This was a retrospective review of 294 children with cerebral palsy reporting number of ITB surgeries per patient, risk of infection for primary and secondary ITB-related procedures, microorganisms responsible and associated factors such as concurrent orthopaedic interventions, medical comorbidities, and subsequent management of ITB-related infections were evaluated.

*Results:* Infection occurred in 28/294 patients (9.5 %) with a 4.9 % rate per procedure. There were 14 acute (within 90 days of surgery) and 14 late infections. The infection risk per ITB procedure was 2.4 %. Risk of Late infection risk over 5 year mean follow-up was 0.95 % per year. Pump removal with acute contra-lateral implantation was the most successful treatment of infections. GMFCS level V and G-tube were the main risk factors for infection. One hundred and thirty three concurrent orthopedic procedures were performed during 277 ITB procedures with no increased risk of infection.

*Conclusion*: ITB in children with CP has a risk of infection which is relatively low and manageable.

*Significance*: It is important to always consider infection as a complication with ITB, with prompt treatment the positive impact of ITB is still possible. It is safe to do concurrent orthopedic procedures with ITB procedures.

**April 16**
IFPOS session
14:20–18:30
VIEUX PORT 300
**IOP4**/14:40–14:45

## Longterm therapy with intrathecal Baclofen improves life quality in children with severe spastic cerebral palsy

Tanja Kraus[1], Kathrin Gegenleitner[1], Martin Svehlik[1], Georg Singer[1], Gerhardt Steinwender[1]

[1]Medical University Graz, Graz, Austria

*Introduction*: Children with severe spastic cerebral palsy (CP) are highly limited in daily life activities causing a reduced quality of life (QoL). This is partly due an increased muscle tone causing pain and contractures. Continuous intrathecal infusion of baclofen (ITB) has been shown to reduce the spasticity of affected patients.

The hypothesis o the present study was that ITB leads to a significant improvement of QoL in non-ambulant children with CP.

*Patients and methods*: QoL was assessed before and after baclofen pump implantation using standardized questionnaires (CP Child, KINDL). Additionally spasticity was evaluated using the modified Ashworth Scale (MAS) at the two time points. Parents were asked whether they would choose ITB treatment for their child again.

*Results:* 13 patients (10 male, 3 female) completed this long-term survey. Mean time between pump implantation and follow up was 60 month (range 12–100). CP Child Score significantly improved from a mean of 60 to a mean of 40 points ($p < 0.05$). Likewise, KINDL showed a significant improvement comparing pre- and post-implantation values (59.4 vs. 60.5 points, $p < 0.05$). MAS markedly decreased from a mean of 3.8 to a mean of 1.7 ($p < 0.05$).

All interviewed caregivers indicated that their expectations have been met; the quality of life of their children improved and would choose ITB treatment again.

*Discussion*: Intrathecal treatment of Baclofen seems to be an excellent method of treatment in children with severe cerebral palsy. Quality of life sustainably improves, parents satisfaction is high and the level of spasticity decreases. Therefore, baclofen treatment can be highly recommended in non-ambulant children suffering from CP.

**April 16**
IFPOS session
14:20–18:30
VIEUX PORT 300
**IOP5**/14:50–14:55

## Chiari osteotomy in spastic cerebral palsy: salvage or reconstruction?

Patricia Fucs[1], Felipe Campos[1], MARCOS Simionato[1], Camila Cavalheiro[1], Helder Yamada[1], Celso SvartMAN[1]

[1]Santa Casa Medical School, Sao Paulo, Brazil

Chiari osteotomy in spastic cerebral palsy: salvage or reconstruction? Felipe Augusto Garcez de Campos, Marcos Cazer Simionato, Camila Maftoum Cavalheiro, Helder H. Yamada, Celso Svartman, Patricia M. de Moraes Barros Fucs

Santa Casa Medical School and Hospitals

*Introduction:* The treatment of the spastic hip deformity in adolescents and young adults with Cerebral Palsy (CP) is based on two ways: reconstruction or salvage. The Chiari osteotomy does an interposition with no articular cartilage, reason for considering as a salvage procedure. The question is if the outcome of the osteotomy in a long-term is satisfactory why it is considered as salvage and not a reconstruction procedure.

*Patients and methods:* From February 1987 to August 2013, 24 patients (24 hips) with spastic hip deformity were submitted to Chiari osteotomy. 2 patients did not return to the last follow-up evaluation. 5 patients were male and 17 female. Functionally 8 patients community ambulators (GMFCS I and II), 4 household ambulators (GMFCS III) and 10 non-ambulators (GMFCS IV and V). The mean age at surgical procedure was 188 months (min 121 and max 302). In all but 3 patients proximal femoral varus, derotation and shortening osteotomy was associated. The mean follow-up period was 98,05 months (min 12 and max 244). Radiographically, on ap view, on preoperative, immediate postoperative and final evaluation were measured: Reimers index, neck-shaft angle and acetabular index. On postoperative was also measured the amount (%) of the medial displacement and the presence of osteoarthrosis on the hip joint.

*Results:* Radiographically all measurements shown statistically significant improvement and maintenance of the correction in the time. The mean medial displacement after the osteotomy was 51,48 %. In 6 cases signs of osteoarthrosis were found, being 4 good clinical result and painless hips; and other 2 patients with fair results, but only one with pain on the hip joint.

Clinically, according to Osterkamp criteria there were 17 good and 5 fair results. Pain was present in 4 cases, being 2 good results and 2 fair. From these 4 cases, 3 were community ambulators (GMFCS II and III) and 1 patient a non ambulator (GMFCS V).

*Conclusion:* Due to the amount of correction obtained with the Chiari osteotomy and the durability of the procedure the authors present a different perspective of the use of the osteotomy, not as a salvage but a reconstructive procedure, in patients with spastic hip deformity in CP.

**April 16**
IFPOS session
14:20–18:30
VIEUX PORT 300
**IOP6**/14:55–15:00

## A descriptive analysis of the upper limb movement during gait in individuals with cerebral palsy

Alice Bonnefoy-mazure[1], Yoshi Sagawa Jr[2], Geraldo de coulon[3], Pierre Lascombes[4], Stephane Armand[5]

[1]University Hospital geneva, geneva, Switzerland
[2]CHRU Besancon, Besancon, France
[3]University Hospital geneva, Geneva, Switzerland
[4]University Hospital of Geneva, Geneva, Switzerland
[5]University Hospital of geneva, Geneva, Switzerland

*Introduction and aim:* Patients with cerebral palsy (CP) are characterised by a large diversity of gait deviations; thus, lower limb movements during gait have been well-analysed in the literature [1]. However, the question of upper limb movements and, more particularly, arm movements during gait has received less attention for CP patients [2]. Thus, the aim of this study was to investigate upper limb movements for a large group of CP patients.

*Patients/materials and methods:* A retrospective search was used, including upper limb kinematic parameters and 92 CP patients [42 females and 50 males, mean ± standard deviation (SD); age 15.2 ± 6.7 years]. The diagnoses consisted of 48 Hemiplegic (HE) and 44 Diplegic (DI). A control group (CG) of 15 subjects was included in the study to provide normal gait data (7 females and 8 males, age 18.4 ± 8.4 years). For the DI patients and control group, 88 arms and 30 arms were analysed, respectively. For the HE patients, 48 affected arms (HE-A) and 48 non-affected arms (HE-NA) were analysed. The kinematic parameters selected and analysed were shoulder elevation angles; elbow flexion angles; thorax tilt and obliquity angles; hand vertical and anterior-posterior movements; and arm angles [3, 4]. Statistical analyses were performed to compare CG with the affected and non-affected upper limbs of HE patients and with the two upper limbs of DI patients.

*Results:* The results showed that HE and DI patients have altered upper limb movements. Moreover, DI patients have greater arm angle, shoulder and elbow movements compared with HE patients. HE patients adopt different shoulder movements between their affected (HE-A) and non-affected arms (HE-NA). Their non-affected arms have no differences with the arms movements of the CG excepted for the range of motion of their arms.

*Discussion and conclusions:* Arm movements have an important biomechanical role during CP gait for stabilisation, compensation and to develop strategies that optimise the gait despite the lower limb level impairments and poor balance control. Thus, the patients move their upper limbs especially as gait deviations are important. These observations confirm that the upper limbs must be integrated into rehabilitation programs to improve inter-limb coordination, to decrease energy expenditure and also to improve gait speed and gait patterns.

**April 16**
IFPOS session
14:20–18:30
VIEUX PORT 300
**IOP7**/15:05–15:10

## Re-application of Pavlik harness for the treatment of DDH

Reiko Murakami[1], Makoto Kamegaya[2], Takashi Saisu[3], Jun Kakizaki[4], Naoto Endo[1]

[1]Div.of Ort.Surg. Niigata Univ., Niigata, Japan
[2]Chiba C & A Orthopaedic Clinic, Chiba, Japan
[3]Chiba Chidren's Hospital, Chiba, Japan
[4]Chiba Children's Hospital, Chiba, Japan

*Introduction*: Pavlik harness treatment is introduced as a useful technique for developmental dysplasia of the hip (DDH) by Pavlik in 1957. Subsequent studies showed the long-term results of the Pavlik harness treatment and preferable outcome to other treatment methods. Therefore, we have expectantly re-applied the Pavlik harness to DDH patients following a 4 week-interval after the failure of the initial 2-week application. The purpose of this study is to clarify the efficacy of the re-application and its long-term result.

*Patients and methods*: Forty two hips in 40 patients treated from 1995 to 2004 were included. The patients were divided into two groups: Group A (22 patients, 24 hips), the initial application was done in our institute; Group B (18, 18), the application was done in other institutes. The re-application was randomly indicated. We assessed the following influence factors of successful reduction at re-application: where the initial application was applied, the presence of the Ortolani sign at the re-application and ultrasonic findings. We also evaluated the duration between the re-application and the reduction and avascular necrosis based on Salter's classification at the final follow-up.

*Results:* The reduction rate was 45.8 % in Group A and 50 % in Group B. A positive click sign at the initial application was found in 90.9 % of patients with successful reduction and in 38.4 % of patients with unsuccessful reduction in Group A. In Group B, a positive click sign before the re-application was found in 22.2 % of those with successful one and 20 % of those with unsuccessful one. The average duration between the re-application and the reduction were 5.7 and 9.0 days, respectively. In the anterior approach of ultrasonic scanning with the Pavlik harness, cartilaginous anlage of the femoral head and supero-medial metaphysis of the femoral neck appeared at the same depth of the posterior acetabular level in all hips with successful reduction during the initial two-week application (Suzuki's type B). There was no avascular necrosis found in patients with successful reduction at the final follow-up.

*Conclusions*: The reduction rate of the re-application was 47.6 %. The re-application of Pavlik harness is safe, simple and beneficial in less cost and less physical and mental stress to patients and their families. This is worthy to try after the failure of first application.

**April 16**
IFPOS session
14:20–18:30
VIEUX PORT 300
**IOP8**/15:15–15:20

### Does failure of closed reduction carry a higher risk of osteonecrosis in subsequent surgery for DDH?

Chia-Hsieh Chang[1], Hao-Che Tang[1], Wei-Chun Lee[1], Hsuan-Kai Kao[1], Wen-E Yang[1]

[1]Chang Gung Memorial Hospital, Taoyuan, Taiwan

*Background:* Osteonecrosis of the femoral head is a major complication in treatment of developmental dysplasia of the hip (DDH). Re-dislocation and secondary surgeries were regarded as risk factors of osteonecrosis. This study aims to clarify whether previous failure of closed reduction a risk factor of osteonecrosis in subsequent surgery.

*Materials and methods:* We retrospectively studied 124 patients treated by open reduction and pelvic osteotomy for unilateral DDH before 3 years old. Twenty-five patients had failed closed reduction before the surgery (secondary surgery group), and the other 99 patients had the surgery as the first treatment (primary surgery group). Osteonecrosis was defined by fragmentation of epiphysis and broadening of femoral neck in the first 3 years after operation. The background data and rate of osteonecrosis were compared between the two groups using t test and Chi square test.

*Results:* Age at surgery and Tönnis grade were not significantly different between the two groups. Fragmentation of epiphysis was observed in 6 of the 25 patients (24 %) in secondary surgery group and 34 of the 99 patients (34 %) in primary surgery group ($p = 0.323$). Rate of neck broadening was 28 % in secondary surgery group and 64 % in primary group ($p = 0.001$).

*Conclusions:* Soft tissue tension could be reduced by previous attempt of closed reduction, and re-dislocation would not carry a greater risk of osteonecrosis in the subsequent open reduction.

**April 16**
IFPOS session
14:20–18:30
VIEUX PORT 300
**IOP9**/15:20–15:25

### Is the transverse acetabular ligament hypertrophied and hindering reduction in developmental dysplasia of hip ?

Soon Hyuck Lee[1]

[1]Korea University Anam Hospital, Seoul, Korea, Republic Of

The transverse acetabular ligament (TAL) in hip joint is in reality a portion of the acetabular labrum.The function of that in adult is not currently well known. But there are many description about TAL in developmental dysplasia of hip. The current literature concur that TAL in developmental dysplasia of hip may become hypertrophied and increased in thickness, thus hindering reduction. Failure of release of that are reported to be one of the major causes of failed open reduction in DDH. The authors observed whether the description in literature about TAL in DDH is valid.

Nine DDH cases treated by arthroscope-assisted open reduction were reviewed. The intraarticular pathoanatomy possibly causing impediment to reduction, especially the shape of TAL were obsesrved. The change after section of TALs was also observed. The intraarticular pathoanatomy was compared with the finding of 3 months-old baby received arthroscopic drainage for septic hip.

The limbus in all 9 hips had hypertrophied, blunted margin causing the narrowing of the acetabular introitus. The shape of TAL in nine DDH was not promonent, hypertrophied or stretched compared with that of the septic hip baby. After section of TAL, the gap of cut margin became widened minimally in 3, slightly in 2, some in 4.

Despite there are no published reports establishing the normal size for TAL, we could not find any evidence of hypertrophy or streching of TAL in DDH, hindering reduction. It appears that the section of TAL did not provide immediate deformation of ring of acetabular labral-chondral complex, inducing substantial widening of acetabular introitus accepting the femoral head. But the section of TAL may result in the loss of hoop stress of the ring of labrum. Considering the viscoelastic nature of labrum that can be helpful in gradually accepting the femoral head deeply after reduction.

**April 16**
IFPOS session
14:20–18:30
VIEUX PORT 300
**IOP10**/15:30–15:35

### Prognostic radiographic factors in developmental dysplasia of the hip following Salter osteotomy

Chia Chang[1], Wen-E Yang[1], Hsuan-Kai Kao[1], Wei-Chun Lee[1], Ken Kuo[2]

[1]Chang Gung Memorial Hospital, Taoyuan, Taiwan
[2]National Taiwan University Hos, Taipei, Taiwan

*Background:* Radiographic parameters for evaluating hip development are altered by Salter osteotomy and their prognostic value required further validation.

*Methods:* The study population involved 63 patients who underwent open reduction and Salter osteotomy for unilateral hip dysplasia were evaluated with Severin classification 10 years later. The initial first year postoperative acetabular index, c/b ratio, head-teardrop distance, and head coverage were compared with final outcome of Severin classification. Significant factors were

*Results:* After follow-up over a mean of 10.8 years, Severin excellent or good results were noted in 54 hips and residual dysplasia in other 9 hips. Greater c/b ratio was significantly associated with later Severin class III hip. Using receiver operating characteristics curve, a c/b ratio >0.72 at 6 months and 1 year postoperatively can predict the possibility of a class III hip in 30 and 60 % of patients, respectively.

*Conclusion:* The c/b ratio was a significant predictor for hip development after Salter osteotomy. The first year postoperative c/b ratio >0.72 can be a warning sign for consideration of further intervention. Acetabular index is not a prognostic parameter to predict outcome after Salter osteotomy. of the acetabulum by

**April 16**
IFPOS session
14:20–18:30
VIEUX PORT 300
**IOP11**/15:35–15:40

## Developmental pattern of the hip in patients with hereditary multiple exostoses

Kwang-Won Park[1], Ji-Ho Lee[1], Han-Ju Kim[1], Hae-Ryong Song[1]

[1]Korea University Guro Hospital, Seoul, Korea, Republic Of

*Background and purpose:* Coxa valga is a common clinical feature of hereditary multiple exostoses (HME). The current study aimed to determine the unique developmental pattern of the hip in patients with HME and evaluate the factors that influence its progression.

*Methods:* Thirty patients (57 hips) with HME were divided into two groups according to the Hilgenreiner epiphyseal angle (HEA). Twenty-two patients (44 hips) including 13 men and 9 women were assigned to group 1 (HEA <25°), and 8 patients (13 hips) including 3 men and 5 women were assigned to group 2 (HEA ≥25°). The mean age at the initial presentation was 6.0 [95 % CI (Confidence Interval), 4.8–7.1] years with 6.8 (95 % CI, 5.9–7.8) years of follow-up in group 1, and 10.4 (95 % CI, 8.8–12.0) years with 5.4 (95 % CI, 3.5–7.3) years of follow-up in group 2. We measured the HEA, neck-shaft angle (NSA), acetabular index angle (AI), center-edge angle (CEA), and migration percentage (MP) for radiographic evaluation.

*Results:* Among the patients, 50 (87.7 %) hips had coxa valga and 27 (47.4 %) hips had abnormal MP (42.1 % were borderline and 5.3 % were subluxated). There was a significant difference in the HEA and NSA between the groups ($p < 0.001$ and $p < 0.05$, respectively). The HEA had a significant effect on the development of the NSA and no correlation was found between the HEA and AI, CEA, and MP.

*Interpretation:* There was a significant relationship between the HEA at the initial presentation and the NSA at skeletal maturity. We should consider guided growth for patients with lower HEA to prevent significant coxa valga deformity with close follow-up.

**April 16**
IFPOS session
14:20–18:30
VIEUX PORT 300
**IOP12**/16:15–16:20

## Hip subluxation and acetabular dysplasia in hereditary multiple exostosis

Hsuan-Yu Chen[1], Kuan-Wen Wu[1], Ken N. Kuo[1], Ting-Ming Wang[1]

[1]NTUH, Taipei, Taiwan

*Purpose:* Hereditary multiple exostosis (HME) commonly occurs in the metaphyses of tibia and femur around the knee. It is transmitted as autosomal dominant with variable penetrance. There were reports that HME occurred in proximal femur can cause anomalies of the hip. It is the purpose of the study to observe the natural history of the hip dysplasia in HME.

*Methods:* From 2002 to 2012, we retrieved 43 patients with HME in our institution. Among them, there were 16 cases with coxa valga and/or acetabular dysplasia. We divided cases into two groups, eight of them received no treatment related to the hip by just observation, and eight of them received tumor excision combined with varus osteotomy or tumor excision only. The retrospective radiographic evaluations included Sharp angle, center-edge angle, neck shaft angle and Reimer’s migration percentage. We retrospectively compared 2 groups for their long term effect of HME. Besides we also want to understand the relationship between HME and family history.

*Results:* In entire surgical group, a successful coverage of the femoral head had been achieved in all radiographic parameters. However, in the observation group, the hip coverage continued to worsen. Among them, 3 patients ended up with severe hip subluxation and degenerative change, which required total hip replacement at a very young age.

*Conclusion:* Hip involvement in patients with HME is relatively rare and usually presented as coxa valga hip dysplasia, and hip subluxation. Due to increased valgus deformity from abnormal growth secondary to osteochondroma, and space occupying osteochondroma at medial side of the femoral neck that displace femoral head laterally. The femoral head often becomes subluxed that resulted in acetabular dysplasia. To prevent this from happening, the timely varus osteotomy and removal of offending osteochondroma are deemed necessary.

**April 16**
IFPOS session
14:20–18:30
VIEUX PORT 300
**IOP13**/16:20–16:25

## Femoroacetabular impingement in hereditary multiple exostoses

Chang Seon Oh[1], Sung Taek Jung[1], Bo Ram Na[1]

[1]Chonnam Natl. Univ. Hosp., Gwangju, Korea, Republic Of

*Purpose:* To study the incidence of femoroacetabular impingement and the radiographic features of pelvic and proximal femoral mass in hereditary multiple exostoses (HME)

*Methods:* Sixty-three patients (thirty-eight males and twenty-five females) with HME were evaluated by radiological analysis. We have divided into femoroacetabular impingement (FAI) group (105 limbs, 83.3 %) and non-FAI group (21 limbs, 16.7 %) based on the head sphericity. On the anteroposterior hip radiographs, we measured the alpha angle, femoral neck-shaft angle, center edge angle, Sharp's acetabular angle, and Reimer's migration percentage. Also, we have checked the hip computed tomography (CT) in twenty-three patients (except 2 limbs for operation), and compared with the results of radiograph. On the other hand, we have evaluated twelve patients (except 1 limb for operation), who were immature patients in longitudinal study.

*Results:* Statistically, significant differences were observed between the FAI and non-FAI group in alpha angle and neck-shaft angle. But there were no significant differences in center edge angle, acetabular angle, and migration percentage. The alpha angle and the neck-shaft angle showed a positive correlation. The mean of alpha angle was 63.7° (±10.1) in CT, and 34 limbs (74 %) had a mass in anterior of femoral head and neck. We were able to find the 2.9 pelvic masses in CT, but 1.8 (61.5 %) masses in radiograph. Based on the longitudinal study, the mean age of abnormal alpha angles discovered was 10.1 years.

*Conclusion:* Asymptomatic FAI were observed commonly in HME, therefore close observation and serial follow-up is needed for prevention of early osteoarthritis and skeletal deformity.

**April 16**
IFPOS session
14:20–18:30
VIEUX PORT 300
**IOP14**/16:30–16:35

## Health-related quality of life, clinical and radiological outcome in 68 patients with LCPD with a mean follow-up of 27 years (15–42)

Yasmin D. Hailer[1]

[1]University Hospital Uppsala, Uppsala, Sweden

*Aim:* 68 patients with unilateral LCPD and a mean follow-up of 28 years (15–42) were examined in order to evaluate health-related quality of life (EQ-5D; EQ-VAS), Harris Hip Score (HHS) and clinical and radiological outcome.

*Results:* There was a correlation of age at onset and range of motion deficit (abduction deficit, r = 0.44**; external rotation deficit, r = 0.36**; flexion deficit, 0.31*; internal rotation deficit, 0.272*). Furthermore, age at onset correlated negatively with HHS (r = −0.41**), the angle of Tönnis (r = −0.31*) and the Stulberg classification (r = −0.41**). A shorter articulo-trochanteric distance (ATD) was correlated with a greater external rotation deficit (r = 0.43**) a higher leg length shortening and a lower score in the HHS (−0.35**). There was a good correlation between the HHS and Stulberg classification (r = −0.62**). The more years with LCPD the worse was the HHS. A subanalysis of patients who underwent either VRO or Salter osteotomy showed lower Scores in health-related quality of life in the group with Salter osteotomy.

*Conclusion:* Age at onset of LCPD is an important factor in the clinical and radiological outcome as well as in the outcome for health related quality of life. ATD influences the range of motion in patients with LCPD and the outcome HHS. The more years with LCPD deteriorate the health-related quality of life. VRO seems to have a favorable impact on health related quality of life in comparison to Salter osteotomy.

**April 16**
IFPOS session
14:20–18:30
VIEUX PORT 300
**IOP15**/16:35–16:40

## Subluxation of the femoral head as a predictor of final outcome in non-operatively treated LCP disease

Hui Taek Kim[1], Tae Hoon Lee[1]

[1]Pusan National University, Busan, Korea, Republic Of

*Purpose:* This study was designed to evaluate the femoral head subluxation related to final outcomes in LCP patients who were treated nonoperatively.

*Materials and methods*: 150 patients with unilaterally affected hips who (1) were between 6.0 and 12.0 years of age at the onset of the disease, (2) underwent nonoperative treatment and (3) were followed up until skeletal maturity, were included in this study. The average age at the time of diagnosis was 7.8 years. Thirteen patients received no treatment, 80 were treated with a brace, and 57 by range-of-motion exercise. All hips were classified by gender, chronologic age, skeletal age and a modified lateral pillar classification. To analyze the factors related to result, we measured the medial joint space widening and superior migration of the femoral head on anteroposterior plain radiographs at the maximal fragmentation stage. Medial joint space widening was defined when the medial margin of the femoral head was located outside (lateral) of the medial 1/3 of the acetabular roof (from the lateral margin of the teardrop to the lateral margin of the acetabular roof). Superior migration of the femoral head was defined when the inferomedial border of the proximal part of the femur was located above the lowest 1/6 of a horizontal band between the inferior margin of the teardrop and the horizontal line which connecting the triradiate cartilage on both sides Final results were classified by a modified Stulberg classification. Statistical analysis was done using the Chi square test and multinomial logistic regression analysis.

*Results:* 68 patients without medial joint space widening or superior migration of the femoral head showed mostly favorable results: Stulberg I and II, 62 (91.2 %); III, 4 (5.9 %); and IV and V, 2 (2.9 %). In contrast, 82 patients with medial joint space widening and/or superior migration of the femoral head had mostly unfavorable outcomes: Stulberg I and II, 6 (7.3 %); III, 41 (50 %); and IV and V, 37 (42.7 %). These differences in outcomes were highly statistically significant (p = 0.001).

*Conclusion*: This study suggests that presence of medial joint space widening and/or superior migration of the femoral head at the fragmentation stage are related to poor outcomes in LCP patients who are treated nonoperatively. For those patients, we carefully consider surgical treatment.

**April 16**
IFPOS session
14:20–18:30
VIEUX PORT 300
**IOP16**/16:45–16:50

## Fixator assisted internal fixation for deformity correction

Sherif Galal[1]

[1]Cairo University, Cairo, Egypt

Correction of deformities by osteotomy is well known. After osteotomy stabilization can be achieved either by internal fixation (K-wires, screws, plates or intramedullary nails), external fixation (unilateral or circular), casting. We asked whether we could accurately correct the deformities based on our preoperative goals for mechanical axis deviation (MAD) using internal fixation after adjusting the correction intraoperatively by a fixator that's removed at the end of surgery.

We identified 26 patients (34 limbs) who underwent corrective osteotomies under image intensifier. We observed accuracy of correction (based on correction of the MAD), duration of surgery, postoperative knee ROM, and complications. Minimum follow-up was 11 months (6–18 months).

We achieved the desired MAD within 10 mm of the goal in 28 of 34 limbs. The Operative time was 86 $\pm$ 34 min per bone. Preoperative and postoperative knee ROMs were similar and there were no major complications.

Fixator-assisted internal fixation combines the accuracy, minimal invasiveness of fixators, with patient compliance and the comfort of internal fixation. Temporary external fixation allows precise correction of the deformity and will be removed at the end of the operation.

**April 16**
IFPOS session
14:20–18:30
VIEUX PORT 300
**IOP17**/16:50–16:55

## Nonproductive arthrocentesis in established septic arthritis of hip in neonates

Soon Hyuck Lee[1]

[1]Korea University Anam Hospital, Seoul, Korea, Republic Of

Delayed diagnosis of septic arthritis of hip persistently haunts neonates. Arthrocentesis provides confirmative diagnosis but may not reveal pus in the established septic arthritis. The aim of this study is to clarify the clinical presentations and the meaning of the dry tab in our case series.

Ten consecutive cases treated by one author between 2000 and 2012 were retrospectively reviewed. There were seven hospital-acquired and three community-acquired infections. The interval from reported symptom onset to surgery was between 2 and 51 days (mean 21 days). All underwent anterior incision and drainage and postoperative immobilization. Before opening the joint capsule, arthrocentesis was attempted to. Functional and radiographic outcomes (mean duration of follow-up: 30 months) were evaluated.

Clinical presentations at referral were pseudoparalysis and irritability by hip motion in all cases, fever in three cases, and swelling of thigh in three cases. Leukocytosis, increased ESR, and increased CRP levels were noted in six, seven, and five cases, respectively. The six cases recieved antibiotic treatment before referral (range 7–51 days, mean 23 days). Antibiotic treatment before referral lowered the incidence of fever or leukocytosis. Simple radiography revealed metaphyseal rarefaction in the proximal femur in seven and with additional periosteal reaction in four cases. In three, no bone changes were found. MRI showed joint effusion or synovial thickening in all cases, subluxation in eight cases, and epiphysiolysis in one case. Extensive surrounding soft tissue edema were noted in seven and additional extraarticular abscess pocket existed in four. Arthrocentesis revealed purulent fluids in three cases and dry tab or scanty serous fluid in seven cases, which had organized, granulation-like tissue in the joint. These seven hips with dry tab showed extensive surrounding soft tissue edema with or without additional extraarticular abscess pocket in MRI. These imaging is suggesting to have association with the drainage after spontaneous rupture of joint capsule. Fortunately, all had stable, congruent joints at the last follow-up radiograph. Among eight of follow-up more than 2 years, two received surgery for acetabular dysplasia and leg shortening. Four is expecting surgery due to length discrepancy and proximal femur deformity.

Clinical presentations and laboratory findings in neonatal septic arthritis of hip were attenuated by previous antibiotic treatment. Arthrocentesis can frequently be negative due to drainage by spontaneous rupture of joint capsule in established septic arthritis. Clinical suspicion should lead to the definite treatment. MRI can provide the diagnostic clues and further informations.

**April 16**
IFPOS session
14:20–18:30
VIEUX PORT 300
**IOP18**/17:00–17:05

## Universal clamp in adolescent idiopathic scoliosis. A 134 patients series jerome sales de gauzy[1], Paul Maisongrosse[1], benjamin plas[1], régis pailhes[1], Aissa Ibnoulkhatib[1], Franck Accadbled[1]

[1]hôpital des enfants toulouse, Toulouse, France

Sublaminar tapes represent in scoliosis surgery an alternative to screws or hooks. Preliminary results demonstrated their efficacy with a low complication rate.

Our aim was to confirm these results in a larger series with longer follow up.

*Material and methods:* One hundred and thirty four patients with idiopathic scoliosis operated on between 2005 and 2010 were included in a retrospective, single center study. There were 20 boys (14 %) and 114 girls (86 %), mean age 15 years (13–20). Curves were classified according to Lenke with 48 type 1, 17 type 2 and 69 type 3. All cases consisted in posterior instrumentation, correction and fusion using a hybrid construct combining 2 cranial hook claws, Universal Clamps in the mid thoracic segment and screws at the low thoracic or lumbar level. Local bone graft was added with Biosorb tricalcium phosphate. Statistical analysis was performed by an independent statistician using Excel and SPSS softwares.

*Results:* Mean follow up was 36 months (24–60). Mean surgical time was 180 min (90–240) and mean blood loss was 324 ml (100–1,000). Average number of Universal Clamps was 4.83 clamps (4–7), for an average 12 levels instrumented (10–15).

*Thoracic curve analysis*

Preoperative Cobb angle was 52.8° (40°–88°) and flexibility 43.2 % (14–100 %).

Postoperative Cobb angle was 14.7° (0°–45°), with 72.9 % average correction (34–100 %).

Two year follow up Cobb angle (134 patients) was 16.5° (0°–45°).

Five year follow up Cobb angle (53 patients) was 15.5° (5°–33°).

Cincinnati Index at latest follow up was 1.78 (0.52–3.69) with 70 % average correction (35–100).

*T5T12 kyphosis analysis*

*Hypokyphosis group (T5T12 < 20°)* (45 patients)

Preoperative Cobb angle was 12.7° (5°–19°). Postoperative Cobb angle was 14.3° (7–25). Latest follow up Cobb angle was 15° (10°–26°).

*Normal kyphosis group (20°–40°)* (82 patients). Preoperative Cobb angle was 27.9° (20°–40°). Postoperative Cobb angle was 19.5° (15–35). Latest follow up Cobb angle was 20.4° (15–35).

*Hyperkyphosis group (>40°)* (7 patients). Preoperative Cobb angle was 49.7° (40–68). Postoperative Cobb angle was 24.5° (13–36). Latest follow up Cobb angle was 24.7° (14–37).

There has been no neurological or septic complication. One patient presented a postoperative pleural effusion which rapidly subsided without treatment. At latest follow up, 17 patients complained of occasional back pain.

*Conclusion:* This study confirms efficacy of sublaminar tapes with similar frontal correction as other fixation implants and improvement or preservation of thoracic kyphosis with no specific complication. The easy implantation technique provides diminution of operative time and blood loss.

**April 16**
IFPOS session
14:20–18:30
VIEUX PORT 300
**IOP19**/17:05–17:10

## Results at skeletal maturity of a fusionless technique. analysis of final correction and complications in 21 patients

Lotfi Miladi[1], Thierry Odent[1], Nejib Khouri[1], Christophe Glorion[1]

[1]Necker Hospital, Paris, France

*Purpose:* Fusionless techniques are associated with an important complication rate. The use of one or two rods stills controversial. We report the results at skeletal maturity of 21 patients operated on for a progressive scoliosis with a single growing rod construct inserted with a mini invasive approach.

*Methods:* Twenty one children with progressive scoliosis from various etiologies (10 idiopathic, 7 syndromic, 4 congenital) were reviewed. Mean age of the patients at index surgery was 10 years (range 7–12 years). The mean follow-up was 5a9 m (range 3a6 m to 7a10 m). Ten patients had arthrodesis at a mean age of 15 years (range 13a8 m to 16 years), and 11 patients still have the rod without arthrodesis at the end of treatment.

The technique relies on a single rod fixed by a proximal claw with 2 supralaminar hooks and 1 pedicle hook, and two pedicle screws distally. An extra-periostal approach was used to insert the implants and the rod was inserted subfascially. The reserve for the rod lengthening was located distally. The principles of rod position, contouring and anchorage was to obtain in the frontal plane a vertical rod parallel to the CSVL, and in the sagittal plane a perfect fitting to the spine profile. Systematic X-ray controls were performed every 6 months and lengthening was performed in case of worsening of the curve more than 10°.

*Results:* A total of 6 complications in 4 patients (19 %): 4 rod breakage and 2 cases of infection that were treated with debridement and antibiotics. No anchor failure was observed. 8 unplanned surgeries were performed.

At last follow-up, mean curve correction was 57 %. Mean interval between two lengthening procedures was 10 months. A proximal junctional kyphosis was observed in 5 cases without clinical symptoms. No autofusion nor fibrosis was noted at the free levels of the instrumented curve when arthrodesis was done for the 10 patients.

*Conclusion:* The single rod technique was found to be safe and effective to maintain curve correction until skeletal maturity. The complication rate was low thanks to respect of the fundamentals biomechanics rules: rod verticality, perfect sagittal contouring and mini invasive approach

**April 16**
IFPOS session
14:20–18:30
VIEUX PORT 300
**IOP20**/17:15–17:20

## Neuromuscular scoliosis and pelvic obliquity. Instrumented surgical treatment without allograft or autologous bone graft from the iliac crest

Juan Couto[1]

[1]FLENI, Buenos Aires, Argentina

Title: Neuromuscular scoliosis and pelvic obliquity. Instrumented surgical treatment without allograft or autologous bone graft from the iliac crest

*Authors*: Duncan, Carlos MD; Segal, Eduardo MD; Couto, Juan MD

*Introduction*: Scoliosis is a common deformity in patients with Cerebral Palsy, especially in non ambulatory patients, and usually develops a Pelvic Obliquity, PO. The incidence is proportional to the neurological impairment, ranging from 5 % in diplegics to 64 % to 74 % in quadriplegics. The aim of this study is to evaluate our results in the posterior instrumented surgical treatment without allograft or bone graft from the iliac crest in cerebral palsy patients, GMFCS V (wheelchairs dependents), with Neuromuscular Scoliosis and Pelvic Obliquity.

*Materials and methods*: In a retrospective study (Level of Evidence III) we've evaluated 29 patients with a diagnosis of Cerebral Palsy and Neuromuscular Scoliosis with Pelvic Obliquity, surgically treated with posterior instrumented arthrodesis, T2/T4 to Pelvis, without allograft or bone graft from the iliac crest. Mean age at surgery was 12 + 8 yo, 9–16 yo. We reviewed preoperative, PreOP, postoperative, POP, and last follow up, LFU, radiographs. The LFU should be in the last 2 years. We measured Pelvic Obliquity, Coronal Cobb, and coronal/sagittal balance.

*Results:* Mean postoperative follow-up was 39.5, 24–112 months. The mean correction of the spinal deformity was 71.4 % in the POP and 66.3 % in the LFU in comparison to the PreOP. The mean correction percentages of POP and LFU in relation to the PreOP data were: Pelvic Obliquity 73.2 % in the POP and 61/% in the LFU. Coronal Balance 33 % in the POP and 66.3 % in the LFU. Sagital Balance 45 % in the POP and 39 % in the LFU. There were no complications such as infections, neurological deficit or death.

*Conclusions*: We didn't found significant differences comparing the loss of correction between our results and the current bibliography which includes allograft and autologous bone graft from the iliac crest. We found lesser complications such as infections or morbidity related. Further studies with more patients and follow up are recommended to evaluate the best treatment for this complex pathology.

**April 16**
IFPOS session
14:20–18:30
VIEUX PORT 300
**IOP21**/17:30–17:35

## Medial patellofemoral ligament reconstruction for recurrent patellar dislocation: a comparison of 2 operative methods

Baxter Willis[1], Stephen Wang[1]

[1]Children's Hospital, Ottawa, Canada

MPFL reconstruction has become a popular technique in the treatment of recurrent patellar dislocation. Several operative methods have been described to reconstruct the medial patellofemoral ligament (MPFL).

*Methods*: We reviewed the results of 2 operative methods (A) Use of autologous quadriceps tendon and (B) Use of a free hamstring graft commonly employed to correct this entity. Preoperative and postoperative Kujala scores for knee function were employed for each operative group. There were 25 patients in the quadriceps tendon group and 20 patients in the free hamstring group and all patients were reviewed at minimum 2 year post surgery.

*Results:* The postoperative Kujala scores for knee function improved significantly and similarly in each group. The recurrence rate (10 %) was higher than expected but resolved with further surgery in all cases.

The major difference between the 2 groups was in complication rate with 3 significant complications in the hamstring graft group which compromised the results. These included 2 patellar fractures in the early postoperative period and 1 post-op wound infection. There were no significant complications in the quadriceps tendon group.

*Conclusions*: MPFL reconstruction can be an effective adjunct in the treatment of recurrent patellar dislocation. The free hamstring technique with patellar drill holes should be approached with caution because of the potential for post-op patellar fracture.

**April 16**
IFPOS session
14:20–18:30
VIEUX PORT 300
**IOP22**/17:35–17:40

## Physeal bar resection with complete transverse osteotomy at the metaphysis and ilizarov external fixation

Hui Taek Kim[1], Tae Young Ahn[1], Kuk Pil Lim[1]

[1]Pusan National University, Busan, Korea, Republic Of

*Purpose:* Asone way to overcome the difficulty of poor visualization of the traditional oblique tunnel or triangular wedge resection techniques, we performed physeal bar resection through a vertical window with complete transverse osteotomy at the metaphysis. We used an Ilizarov external fixator to fix the short fragment having a physis and to correct associated angular deformity and leg length discrepancy (LLD). However, there is concern that periosteal elevation and its stretching during the bar resection procedures may deter callus formation.

*Materials and methods:* We treated 10 cases of partial epiphyseal closure (distal femur 6, proximal tibia 2, distal tibia 2). Causes of epiphyseal closure were trauma (8), congenital anomaly (1) and infection sequelae (1). Physeal bars were central in 8 cases and mixed in 2, and mean area of the bar was 12.1 %. Mean angular deformity measured by mechanical axis before surgery was 14.3° (valgus knee and varus ankle deformity) in 8 patients and affected legs averaged 17.5 mm shorter than the normal sides. Mean patient age at the time of surgery was 9.9 years and mean follow up period was 31 months. Healing index (HI: days/cm) and external fixator index (EFI: days/cm) were calculated. Final results were evaluated by a modified Williamson-Staheli classification based on angular deformity and LLD.

*Results:* The mean HI was 60.2 and EFI was 70.5 in 8 cases. One patient had delayed consolidation and required a bone graft. In 7 skeletally mature patients, 4 had a valgus mechanical axis (mean 2.2°) while 3 had varus (mean 2.3°). Affected sides averaged 5.9 mm longer than contralateral side in 3 patients and 5.8 mm shorter in 4. Three skeletally immature patients had a mean of 1.2° valgus mechanical axis. Affected legs 6.2 mm shorter than the normal legs in 2 patients and 6 mm longer in 1. One patient had recurrence of bar formation and had additional operation. Final results of bar resection were 8 satisfactory and 2 fair.

*Conclusion:* A direct approach through a vertical window with complete transverse osteotomy provides a good surgical field for resection of a central or mixed physeal bar. Angular deformity and LLD can be corrected satisfactorily with the Ilizarov external fixator. Callus formation may be slightly delayed.

**April 16**
IFPOS session
14:20–18:30
VIEUX PORT 300
**IOP23**/17:45–17:50

## Duration of spica cast treatment for femoral shaft fractures: a retrospective study

Joi Chen[1], Dale Blasier[1]

[1]Arkansas Children's Hospital, Little Rock, AR, United States

*Introduction:*. In children ages 5 and younger, reduction and immobilization in a spica cast is standard treatment. However, spica cast treatment can be a hardship for patients and families. If this period of immobilization could be lessened, it would be beneficial. Currently, the recommended duration of immobilization is 5–12 weeks. The goal of this retrospective study is to determine if there is a difference in outcome for patients immobilized for 4 weeks or less compared to those immobilized for longer than 4 weeks.

*Methods:* We reviewed the charts and radiographs of 57 patients with femoral shaft fractures presenting between January 2009 and December 2013 treated with spica cast immobilization. Patients were excluded with open fractures, polytrauma or additional debilitating conditions. Patients were divided into two groups: those who were treated for 4 weeks or less (≤28 days) and those treated for greater than 4 weeks (>28 days). Patients were screened for refracture, shortening and changes in angular alignment between the time of cast removal and the next taken radiograph. Episodes of unusual pain were determined by chart review. We felt that an unsatisfactory result (suggestive of inadequate period of immobilization) would include refracture, increase in angulation >5°, increase in shortening >5 mm, or subjective persistent or unusual pain that warranted phone calls to clinic, unscheduled return to clinic, or trips to the emergency room that have been documented in the medical records.

*Results:* There were 36 patients immobilized ≤28 days. None had refracture, shortening >5 mm, increase in angulation >5°, or unusual pain. There were 21 patients immobilized >28 days. None had refracture, or shortening >5 mm, one had increase in angulation >5°, and none had unusual pain. The patient with increased angulation had no clinical consequence and required no treatment.

*Conclusion:* Patients immobilized for 4 weeks or less did just as well as those immobilized longer. This study suggests that shorter periods of immobilization may be effective for femur fracture in kids, thus minimizing hardships of children and their families associated with spica cast treatment.

**April 16**
IFPOS session
14:20–18:30
VIEUX PORT 300
**IOP24**/17:50–17:55

## Analysis of clinical management of femoral fracture in neonates

DAHUI WANG[1], Ruixue MA[1]

[1]Fudan University, Shanghai, China

*Objective:* To analyze clinical treatment and outcome of twenty femoral fractures in the neonates and explore the effective measure.

*Methods:* The history records and X-ray films of neonatal femoral fractures during the period from January,2003 to Decemenber, 2013 were reviewed. They included of twelve boys and eight girls. Eighteen fractures of the femur occurred in delivery process. Fourteen fractures occurred in cesarean section and four during vaginal delivery. In these cases, thirteen were oblique and seven transverse, ten occurring at the proximal, eight middle and two distal. No damnification of blood vessel and nerve. No diagnosis of shaft of bone epiphysis. All of them were treated conservatively. Median duration of hospital stay was 16 days.

*Results:* All of them had the treatment except two giving up treatment. Analyzed the clinic treatment and X-ray, all fractures achieved clinical union within 3 weeks. The average angular and shortening deformity were 38°and 0.7 cm. Among them, seventeen patients were followed up for 2 months to 6 years. The remaining deformities were correctedand remodelled during the period of growth and development. They could walk with normal gait, and range of motion of the hip and knee joint were normal. No nonunion happened.

*Conclusions:*The femoral fractures in the neonates usually are caused by trauma during cesarean and falling off and difficult to get anatomical reduction with closed methods. The femur of the neonate is of great potential of plasticity, and the union period is usually short, and few complication can occur, and prognosis is good. The treatment should be simple, safe and effective conservative way. The popular accepted criteria of children's femoral fracture should be enlarged in neonates. The use of repetitively close or open reduction to obtain anatomical reduction must be avoided.

**April 16**
IFPOS session
14:20–18:30
VIEUX PORT 300
**IOP25**/18:00–18:05

## The prognostic value of fracture level in the treatment of gartland type iii supracondylar humeral fracture in children

Seungcheol Kang[1], Minjae Kam[1], Faisal Miraj[2], Soo-Sung Park[1]

[1]Asan Medical Center, Seoul, Korea, Republic Of
[2]Fatmawati General Hospital, Jakarta, Indonesia

*Introduction:* Although the surgical prognosis of Gartland type III supracondylar humeral fracture (SCHF) is satisfactory, some patients experience troubling limited or delayed recovery after surgical treatment. We proposed a new subclassification system of type III SCHF that is based on the fracture level, and assessed its prognostic value under the hypothesis that fracture lines distal to the isthmus of the humerus (i.e., low fracture level) would associate with worse surgical outcomes.

*Methods:* We retrospectively reviewed 230 children who underwent closed reduction and percutaneous pinning (CRPP) for their Gartland type III SCHFs. The clinico-radiological characteristics and surgical outcomes (elbow range-of-motion [ROM] recovery, postoperative angulation, and the final Flynn grade) were recorded. Multivariate analysis was employed to identify prognostic factors that influenced these outcomes, including fracture level.

*Results:* Multivariate analysis revealed that low fracture level associated significantly with poor prognosis in terms of elbow ROM ($p < 0.001$), angulation ($p = 0.001$), and Flynn grade ($p = 0.003$). An older age was another poor prognostic factor for ROM recovery ($p = 0.027$).

*Conclusion:* Fracture level was an independent prognostic factor for poor outcomes in children who underwent CRPP for Gartland type III SCHF. This is the first study introducing a new subtype classification system that can be used to determine the prognosis of surgery.

**April 16**
IFPOS session
14:20–18:30
VIEUX PORT 300
**IOP26**/18:05–18:10

## Traumatic separation of the distal ulnar physis in children: A new classification for displaced volar-flexion injuries

Pen-Gang Cheng[1], Matthew N. Wang[1], Wei-Ning Chang[2]

[1]Kuang-Tien General Hospital, Taichung, Taiwan
[2]Kaohsiung Veterans General Hos, Kaohsiung, Taiwan

*Objectives:* To propose a new fracture classification according to the direction of epiphysis displacement and to compare clinical findings and surgical outcomes between these subtypes.

*Patients/participants:* Twelve adolescents (mean age 13.4 ± 1.3 years) who experienced separation of the distal ulnar physis were identified from the pediatric trauma database.

*Intervention:* Closed reduction was attempted for all injuries. If a satisfactory alignment could not be achieved, an open reduction was performed.

*Main outcome measurements:* The clinical outcome was evaluated with Mikic's criteria. The impacts of fracture patterns and locations of wrist abrasions on treatment decisions and clinical outcomes were tested with Fisher's exact tests (unadjusted) and logistic regression analyses (adjusted for age and gender) with the bootstrap method. Five orthopedic surgeons used the new classification and the reproducibility was tested with multi-rater kappa.

*Results:* The injury patterns included 6 dorsally-tilted distal ulnas (type I) and 6 volarly-tilted distal ulnas [type II-A (n = 1), type II-B (n = 3), and type II-C (n = 2)]. All type I fractures were successfully treated with closed reduction. Five of six cases with type II injuries failed closed reduction due to entrapment of the extensor carpi ulnaris tendon in the fracture site. Eleven of the patients' outcomes were excellent. One patient with a type II-C injury experienced ulnar growth arrest. The multi-rater kappa for the new classification equals to 0.94, and the $p$ value is less than 0.001.

*Conclusions:* The majority of volar-flexion injuries require surgery to reduce the entrapped soft tissue. This new classification is easy to understand with a good inter-rater reproducibility. It is useful in identifying the injury mechanism and correlated with the likelihood of open reduction.

**April 16**
IFPOS session
14:20–18:30
VIEUX PORT 300
**IOP27**/18:15–18:20

## Early experience with bone scan spect ct in adolescents with complex problems in the foot and ankle region

Ole Rahbek[1], Marina Easty[1], Lorenzo Biassoni[1], Deborah Eastwood[1]

[1]Great Ormond Street Hospital, London, United Kingdom

*Aim:* Adolescents, particularly those with proven foot pathology, may develop unexpected foot pain during periods of changing growth rates. It can be difficult to differentiate between a functional and organic aetiology and between pain arising from old/treated pathology versus new pathology. The aim of this retrospective study was to review our preliminary experience on bone scan with SPECT CT in the assessment and management of the child with complex, atypical foot/ankle pain.

*Methods:* We reviewed the notes and imaging of 13 patients (14 symptomatic feet) with complex foot and ankle pain, referred to our tertiary referral centre between 2009 and 2014. All patients had plain films and 9/14 feet had undergone MRI (1 foott had CT). Bone scanning was performed with a dual head Siemens Symbia T2 SPECT/CT gamma camera. Planar blood pool and delayed static images of the feet, with SPECT/CT images of both feet and ankles, were acquired. The CT settings in children <8 year were 80 kVp/30 mAs; and in those >8 year CT 80 kVp/50 mAs. The added value of this investigation was established during a discussion between the radiologists and the orthopaedic surgeons. The underlying diagnoses were: tarsal coalition (n = 3), non-specific inflammation (n = 2), spastic diplegia (n = 4), clubfeet (n = 2), chronic regional pain syndrome, (n = 1), multiple hereditary exostosis [1], accessory navicular bone [1]. In all feet The cause of current symptoms was unclear in all feet.

*Results:* The mean age at investigation was 13 year (range 9–17), 11/13 patients were female. Four feet had undergone previous surgical treatment. Bone scan SPECT/CT added clinical value versus x-rays and MRI/CT in 11/14 feet. In 5 feet it prompted surgical management (fusion, screw removal, coalition excision, arthrodesis). In 3 feet it showed unexpected areas of mechanical stress, thus changing management. It excluded other pathologies in 2 feet. It directed steroid injections to the area of active mechanical stress, with subsequent clinical benefit, in 1 foot. Bone scan SPECT/CT was valuable in 4 post-surgical feet with metal work, where MRI would have been difficult. In 4/14 feet SPECT CT confirmed the diagnosis without adding significant clinical information.

*Conclusion*: Bone scan/SPECT CT localizes areas of soft tissue inflammation and high/low bone turnover, often in a more clinically relevant way than MRI.

*Significance:* In children with complex foot pathology and unclear symptom aetiology, SPECT CT allows identification of areas of physiological/pathological stress allowing both conservative and surgical treatment to be focused appropriately.

**April 16**
IFPOS session
14:20–18:30
VIEUX PORT 300
**IOP28**/18:20–18:25

## Health related quality of life in children with flexible flatfeet: a cross-sectional study

Alpesh Kothari[1], Julie Stebbins[2], Zavatsky Amy[1], Tim Theologis[1]

[1]Oxford University, Oxford, United Kingdom
[2]Oxford Gait Laboratory, Oxford, United Kingdom

*Introduction:* The effect of paediatric flexible flatfeet (FF) on health related quality of life (HRQOL) has not been investigated. In this prospective cross-sectional study, the HRQOL of children with FF was compared to those with neutral feet (NF) using two validated measures. We hypothesised that reduced HRQOL would be observed in children with FF. As it is often reported that parents overestimate the severity of symptoms their child with FF experiences, the reliability of parents' perceptions of their child's symptoms was also investigated.

*Methods:* Ninety-five children (aged 8–15) were prospectively recruited from the orthopaedic clinic and community. Foot posture was classified on the basis of objective, measurable criteria. Forty-eight children were classified as having FF and 47 as having NF. The body mass index (BMI) of each child was calculated. Each participant completed The Oxford Ankle Foot Questionnaire for Children (OxAFQ-C) and Pediatric Quality of Life Inventory (PedsQL™ 4.0). Proxy questionnaires were also completed by participants' parents. Reliability of parent and child questionnaire scores were assessed using the intraclass correlation coefficient (ICC) and Student's $t$ test. Differences in HRQOL between PFF and TDF were assessed using the Student's t-test.

*Results:* There was no significant difference in either age or BMI between the groups, but there was a greater proportion of females in the FF group (p = 0.01). HRQOL scores were independent of gender. There was fair to excellent reliability (ICC 0.60–0.87) between parent and child questionnaire domain scores for all questionnaire domains except for the PedsQL™ 4.0 emotional and social domains which were poor (ICC = 0.38). There was a tendency for parents to overestimate the impairment of the child in the FF group, most marked in PedsQL™ 4.0 physical domain (p = 0.026). FF children demonstrated clinically significant decreased HRQOL compared to NF children. This was most marked in the OXAFQ_C physical domain (FF = 63.45 % vs NF = 83.24 %, $p < 0.001$).

*Discussion:* Although parents may overestimate their child's impairment, children with FF still have significantly impaired HRQOL when compared to children with NF. The impairment can be as severe, or worse, than published HRQOL for acutely and chronically unwell children1. As such FF cannot be regarded as just a benign normal variant. Consideration of symptom profile and HRQOL should be integral to the assessment and management of FF.

Open Access

This article is distributed under the terms of the Creative Commons Attribution License which permits any use, distribution, and reproduction in any medium, provided the original author(s) and the source are credited.


References

1. Varni Med Care 2001 39 8 800 812 10.1097/00005650-200108000-00006 11468499
